# A Difficult and Rare Case of Warfarin Refractory Antiphospholipid Syndrome Presenting With Catastrophic Antiphospholipid Syndrome Complicated by Gastrointestinal Bleeding

**DOI:** 10.7759/cureus.17106

**Published:** 2021-08-11

**Authors:** William Lim, Amandeep Kaur, Foma Munoh Kenne, Maxim Shulimovich

**Affiliations:** 1 Internal Medicine, Richmond University Medical Center, Staten Island, USA; 2 Hematology and Medical Oncology, Richmond University Medical Center, Staten Island, USA; 3 Hematology and Medical Oncology, Brooklyn Cancer Care, Brooklyn, USA

**Keywords:** catastrophic antiphospholipid syndrome (caps), warfarin refractory aps, cardiac arrest, enoxaparin sodium, gastrointestinal bleeding

## Abstract

Antiphospholipid syndrome (APS) is managed with warfarin for secondary prophylaxis in patients who have had a thrombotic event in the past. Warfarin has been deemed superior to novel oral anticoagulants in preventing venous and arterial thrombosis in conjunction with aspirin. The catastrophic variant of APS (CAPS) is very rarely reported, especially in those who have been on a therapeutic dose of warfarin therapy. We present a rare case of CAPS in a patient with a history of APS who had been on a therapeutic dose of warfarin along with aspirin therapy.

The patient is a 70-year-old male with APS diagnosed 30 years prior when he presented with a pulmonary embolism; aspirin was added to warfarin two years ago when he had a cerebrovascular accident (CVA). He presented to the hospital with acute onset right-sided weakness and aphasia, left lower extremity pain. He had ischemic CVA, acute deep vein thrombosis (DVT), acute renal failure with a creatinine of 2.8, anemia with hemoglobin of 3.8, gastrointestinal bleed (GIB) on EGD, with INR of 3.48 cardiolipin IgM of >140g/L. He was transfused packed red blood cells, fresh frozen plasma, and provided Vit K. Subsequently, he had a cardiac arrest and was intubated and placed on a mechanical ventilator. Given simultaneous multiorgan involvement, acute arterial and venous thrombosis, the patient was diagnosed with CAPS. The patient was started on high-dose dexamethasone, intravenous immunoglobulin (IVIG), and underwent plasma exchange with significant improvement in symptoms, laboratory parameters; and was extubated with near normalization of his speech and motor deficits. He was discharged on enoxaparin and prednisone with sustained clinical improvement two months following discharge.

This patient was on the recommended treatment for APS. However, he had presented with a CAPS. This is the first reported case of warfarin refractory CAPS. This case highlights that there might be a subgroup of the population in whom warfarin is not an effective form of treatment modality for an unknown cause, and in fact, it could potently expose a patient to the adverse events related to warfarin therapy as it did in our patient who had significant GIB. This case also highlights the uncommon scenario of spontaneous CAPS with no inciting event as previously reported in the literature, such as infection, recent surgeries, or trauma.

## Introduction

Antiphospholipid syndrome (APS) is a complex disease and can present with a wide spectrum of clinical manifestations. Catastrophic APS (CAPS) is a rare, life-threatening variant of classic APS, occurring in about 1% of patients with APS and has a mortality of nearly 50% [1].

We herein report a case of warfarin refractory CAPS complicated by gastrointestinal bleeding with improvement in symptoms after treatment with plasmapheresis, intravenous immunoglobulin (IVIG), and steroids. This is the first reported case of warfarin refractory CAPS.

## Case presentation

A 70-year-old male with a past medical history of APS for 30 years on warfarin and aspirin, deep vein thrombosis (DVT) in the right lower extremity, pulmonary embolism, prior ischemic stroke two years ago who had presented to the emergency department with new-onset right-sided weakness and expressive aphasia. The patient had no recent history of surgery, infection, or trauma and had been compliant with aspirin and warfarin therapy for APS. On examination, his vital signs included: temperature of 98.5 F, blood pressure of 105/69 mmHg, pulse rate of 87 bpm, and oxygen saturation of 98% in room air. Right-sided weakness was noted with motor power of ⅗ in both upper and lower extremities along with bilateral lower extremity edema. Laboratory studies were notable for the following: hemoglobin of 3.8 g/dL, white blood cell count of 7.3 k/µL, platelet count of 164 k/µL, creatinine of 2.8 mg/dL (baseline creatinine ≅ 1.5 mg/dL), international normalized ratio (INR) of 3.48, prothrombin time (PT) of 34.7 secs, activated partial thromboplastin time (APTT) of 81.4 secs, fibrinogen was 360 mg/dL, and total bilirubin was 0.3 mg/dL. MRI brain revealed small areas of cortical thromboembolic infarcts of the left parietal region. Venous duplex of lower extremity showed new blood clots in the left common femoral and left proximal superficial femoral vein. An echocardiogram showed no patent foramen oval with normal ejection fraction. The patient was started on a pantoprazole drip for GIB, received 4U of packed red blood cells (PRBC) with inadequate response from Hb 3.8 to 5.6. Warfarin was held since admission, and the decision was made to start vitamin K 5 mg PO daily and transfuse 1 unit of fresh frozen plasma. 4-Factor recombinant was held due to the new-onset cerebrovascular accident (CVA), new DVT in the setting of antiphospholipids (aPLS). The patient had inferior vena cava filter placement.

By day 4, the patient’s hemoglobin increased to 8.5 g/dL, and INR normalized after Vitamin K supplements and 9 U PRBC. Esophagogastroduodenoscopy (EGD) showed a pre-pyloric fold gastric ulcer without active bleeding. Colonoscopy showed melena throughout the large colon without any sight of active bleeding. A bleeding scan was obtained, which showed no evidence of GIB. The patient was noted to have a decrease in platelet count since day 2, with a nadir of 38 k/µL on day 4. A total of four units of platelets were transfused with an inadequate response. The patient was then started on high-dose dexamethasone (40 mg intravenous daily), together with IVIG 1 g/kg daily to prevent further complement-mediated peripheral destruction as C3 and C4 were significantly low. Anti-anticardiolipin IgG and IgM antibodies were elevated to >150 g/L. Antinuclear antibody, rheumatoid factor, and anti-double-stranded antibody, and ADAMS-TS 13 were all negative. Despite all interventions, the patient’s neurological status did not improve. Subsequently, he had a cardiac arrest return of spontaneous circulation achieved in three minutes.

Given APS history with a new ischemic CVA, cardiac arrest, acute DVT, acute kidney injury, and high cardiolipin antibodies, the patient was started on plasmapheresis for CAPS. After two sessions of plasmapheresis, the patient’s platelet level started to increase, hemoglobin stabilized, and his neurological status returned to baseline, and he was subsequently extubated. Full-dose anticoagulation with low molecular weight heparin enoxaparin 1 mg/kg was started. Repeat EGD and colonoscopy did not show any active bleeding. The patient was discharged with enoxaparin 1 mg/kg and prednisone two months later, and the patient was found to be doing well, with no re-occurrence of clots or bleeding.

## Discussion

APS is a clinical autoimmune syndrome characterized by venous or arterial thrombosis and/or pregnancy morbidity in the presence of persistent laboratory evidence of aPL antibodies [2]. An aPL antibody includes IgG and IgM antibodies to cardiolipin and beta2-glycoprotein (GP) I and a functional assay for the lupus anticoagulant (LA) phenomenon [3].

Secondary thrombosis prevention with long-term anticoagulation is the mainstay of therapy for APS patients with a history of thrombosis, given the high rate of recurrent thrombosis in these patients. Warfarin remains the drug of choice for the prevention of secondary thrombosis prevention in patients with APS since direct oral anticoagulants (DOACs) are shown to be less effective than warfarin for thrombosis prevention [4-7]. Recurrent thromboembolism in an individual with APS treated with an anticoagulant is rare. There are a few possible interventions for patients who have a recurrent thrombotic event while receiving anticoagulation. For patients with the lower end of the target INR, one approach is to increase the target INR from 2-3 to 3-4 using high-intensity warfarin, although there is no methodologically rigorous evidence to support this strategy [8,9]. And for patients with a higher end of target INR, the approach is to switch anticoagulation from warfarin to low molecular-weight heparin (LMWH) [10]. Since our patient's INR is above 3, warfarin was switched to low molecular weight heparin to prevent further thromboembolism.

A small subset of patients with APS has a widespread thrombotic disease with multiorgan involvement, called "catastrophic APS." Diagnostic criteria have been established for a catastrophic syndrome which includes the involvement of three or more organs, systems, and/or tissues manifesting simultaneously or in less than a week. Also, laboratory confirmation of the presence of aPL antibodies (LA, anticardiolipin antibodies, and/or anti-beta2-GP I antibodies) [11].

Our patient had multi-organ involvement, including new DVT and CVA on supratherapeutic INR, acute renal failure, GI bleed, and a cardiac arrest, with high titers of Cardiolipin IgM and IgG antibodies. Thus, he met the criteria for CAPS and had an excellent response to plasmapheresis and steroids.

Observational data show that plasmapheresis improves survival in patients with CAPS, although it has never been investigated in a randomized trial [12-14]. The theory behind the use of plasma exchange in patients with CAPS is based upon the hypothesis that aPL antibodies may be the prime mediators in thrombosis. Plasma exchange can rapidly remove IgG and IgM, which have half-lives of 22 and five days, respectively. This is supported by a clear decline in IgM cardiolipin level after plasmapheresis and improvement in patient symptomatology and hematological parameters.

High-dose systemic corticosteroids and IVIG are also a part of the treatment of APS [15]. There is no evidence that IVIG alone improves survival. Still, combination therapy of IVIG and plasmapheresis is shown to be more effective for severe CAPS cases and associated thrombocytopenia. In a systematic review of 342 case reports involving patients with CAPS found triple therapy with anticoagulation, glucocorticoids, and either plasma exchange, IVIG, or both has shown to have mortality benefits compared to the treatment strategies that did not use plasma exchange, IVIG, or both [16]. Therefore, in the above-mentioned case of CAPS, the patient has been treated with steroids, IVIG, and plasmapheresis, followed by anticoagulation.

## Conclusions

We discussed a challenging case of warfarin refractory CAPS, which is complicated by gastrointestinal bleeding. The case highlights and adds to a growing body of literature regarding CAPS and illustrates the importance of recognizing the rare clinical entity of so-called CAPS. CAPS is a rare condition, but it has a very high mortality rate; therefore, proper caution needs to be exercised to initiate timely intervention and avoid complications.
